# Habitat-associated detection of *Toxoplasma gondii* and *Sarcocystis* spp. in Cetaceans from the Brazilian coast

**DOI:** 10.1007/s11259-026-11324-y

**Published:** 2026-06-11

**Authors:** Thalita Faita, Lara Borges Keid, Natalia Silvestre-Perez, Gláucia Pereira de Sousa, Geovana Carolaine Ramos Thomé, Beatriz de Magalhães Ceron, Samira Costa-Silva, Cristiane Kiyomi Miyaji Kolesnikovas, Pedro Volkmer de Castilho, Fernanda Loffler Niemeyer Attademo, Fábia de Oliveira Luna, Angélica Maria Sánchez-Sarmiento, Raquel Beneton Ferioli, Vitor Luz Carvalho, Aline Ramos Souza, Marta Jussara Cremer, Jenyffer Vierheller Vieira, Adriana Castaldo Colosio, Milton César Calzavara Marcondes, Vanessa Lanes Ribeiro, Carolina Pacheco Bertozzi, Renata Hurtado, Caroline Freitas Pessi, João Carlos Gomes Borges, Arícia Duarte-Benvenuto, Jennifer Morossino, Paula Lima Canabarro, José Luiz Catão-Dias, Rodrigo Martins Soares

**Affiliations:** 1 Universidade de São Paulo, Pirassununga, São Paulo, Brazil; 2 Universidade de São Paulo, São Paulo, Brazil; 3 Instituto Chico Mendes de Conservação da Biodiversidade, Santos, São Paulo, Brazil; 4 Associação R3 Animal, Florianópolis, Santa Catarina Brazil; 5 Universidade do Estado de Santa Catarina, Laguna, Santa Catarina Brazil; 6 Universidade Federal Rural do Semi-Árido, Mossoró, Rio Grande do Norte Brazil; 7 Instituto Argonauta para a Conservação Costeira e Marinha, Ubatuba, São Paulo, Brazil; 8 Associação de Pesquisa e Preservação de Ecossistemas Aquáticos, Caucaia, Ceará Brazil; 9 Universidade da Região de Joinville, São Francisco do Sul, Santa Catarina, Brazil; 10 Instituto Baleia Jubarte, Caravelas, Bahia Brazil; 11 Instituto Biopesca, Praia Grande, São Paulo, Brazil; 12 Universidade Estadual Paulista (UNESP), São Vicente, São Paulo, Brazil; 13 Instituto de Pesquisas de Cananéia, Cananéia, São Paulo, Brazil; 14 Fundação Mamíferos Aquáticos, Recife, Pernambuco Brazil; 15 Universidade Federal da Paraíba, Rio Tinto, Paraíba, Brazil; 16 Centro de Recuperação de Animais Marinhos, Rio Grande, Rio Grande do Sul Brazil; 17 Departamento de Medicina Veterinária Preventiva e Saúde Animal, Faculdade de Medicina Veterinária e Zootecnia, Universidade de São Paulo, Av. Duque de Caxias Norte 225, Pirassununga 13635-900 São Paulo, Brasil

**Keywords:** *Toxoplasma gondii*, Sarcocystidae, *Sarcocystis neurona*, Marine mammals, Protozoan infections, Terrestrial-to-marine pathogen transmission.

## Abstract

Cetaceans are sentinels of marine ecosystem health and are increasingly exposed to protozoal pathogens. This study investigated the occurrence and molecular identity of Sarcocystidae protozoa in 159 stranded cetaceans representing 21 species along the Brazilian coast. Molecular analysis based on PCR amplification and sequencing of the sarcocystid internal transcribed spacer 1 (ITS1) region revealed infections in 14 individuals, showing a clear habitat-associated distribution. *Toxoplasma gondii* and *Sarcocystis neurona* were detected in nine coastal cetaceans, consistent with transmission from terrestrial definitive hosts via land-to-sea runoff. In contrast, a distinct *Sarcocystis* lineage consistently reported in cetaceans and pinnipeds was identified in six pelagic individuals. Analyses of mitochondrial cytochrome c oxidase subunit I and 18S rDNA markers revealed close similarity to species reported in avian definitive hosts, suggesting a life cycle involving marine or pelagic birds. This ecological segregation reflects differences in parasite transmission pathways and host–habitat interactions. This study provides the first report of *S. neurona* in Brazilian cetaceans and the first global record of this parasite in Guiana dolphin (*Sotalia guianensis*). Overall, these findings emphasize the role of habitat use in shaping infection patterns and reinforce the importance of a One Health framework to understand land–sea and potentially marine-specific pathogen transmission, as well as its implications for marine wildlife health and conservation.

**Supplementary Information:**

The online version contains supplementary material available at https://doi.org/10.1007/s11259-026-11324-y.

## Introduction

Cetaceans play a crucial ecological role as sentinels of marine, coastal, and riverine ecosystems, reflecting environmental health conditions. In Brazil, the suborder Odontoceti comprises 38 species and Mysticeti include nine species (Monteiro-Filho et al. 2021). Several of these species are currently threatened and face multiple anthropogenic pressures, including habitat degradation, pollution, vessel traffic, and bycatch (IUCN, 2025).

Beyond these threats, cetaceans are susceptible to infectious diseases, including protozoa of the phylum Apicomplexa, particularly members of the family Sarcocystidae. These parasites can establish tissue cysts, often resulting in chronic and asymptomatic infections, although acute disease may cause severe lesions such as encephalitis, pneumonia, and hepatitis (Dubey et al. 2015). Transmission dynamics in marine environments remain incompletely understood, but contamination of aquatic systems by oocysts and sporocysts shed by terrestrial definitive hosts (felids for *T. gondii* and didelphid opossums for *S. neurona*) via freshwater runoff is widely accepted (Fayer et al. 2004).

Considering the ecological importance of cetaceans and the potential land-to-sea transmission of sarcocystid parasites, this study investigated the occurrence and molecular identity of Sarcocystidae in cetaceans stranded along the Brazilian coast.

## Materials and methods

Field activities and sample collection were conducted under federal permits issued by the Ministry of the Environment and Climate Change and the Chico Mendes Institute for Biodiversity Conservation (ICMBio), through the Biodiversity Information and Authorization System (SISBIO; permits 75348-2, 45568-1, 55433-1) and registered in the National System for the Management of Genetic Resources and Associated Traditional Knowledge (SISGEN; registration ADA22DD). The study was carried out within the Beach Monitoring Projects of the Santos and Potiguar Basins, conducted by Petróleo Brasileiro S.A. (Petrobras) under environmental licensing by the Brazilian Institute of Environment and Renewable Natural Resources (IBAMA), Ministry of the Environment and Climate Change (License ABIO No. 640/2015 and 1169/2019). All procedures were approved by the Ethics Committee on Animal Use of the School of Veterinary Medicine and Animal Sciences, University of Sao Paulo, Brazil (CEUA-FMVZ; protocol 2016020817).

This study included 159 deceased cetaceans from 21 species stranded along the Brazilian coast between 2004 and 2022, spanning a latitudinal range from approximately − 2.81 to − 29.04. Animals of both sexes and different age classes were represented, including coastal, pelagic, and migratory species (Table 1). Coastal species included taxa with more resident or nearshore habits, whereas pelagic species were generally represented by wide-ranging oceanic cetaceans with broader movement patterns. Carcass decomposition codes ranged from fresh (code 2) to advanced autolysis (code 4) (Geracy and Lousbury 2005). Necropsies were performed on site or near the locations where the animals were found, by collaborative teams from the associated regional research centers. During postmortem examination, organ samples were collected for molecular investigation of parasitic infections. Whenever possible, fragments (~ 2 cm³) from internal organs with gross lesions were aseptically obtained. Samples were individually identified and stored at − 80 °C until molecular analysis.

**Table 1:** Detection of Sarcocystidae by nested PCR targeting the ITS1 region (nPCR-ITS1) and corresponding parasite species identified in cetaceans from the Brazilian coast

Cetacean species	Habitat type	Individuals tested	nPCR-ITS1 positive (*n*)	T. gondii (*n*)	Sarcocystis sp. (*n*)	S. neurona (*n*)
*Pontoporia blainvillei*	Coastal	28				
*Sotalia guianensis*	Coastal	47	7	5		2
*Delphinus delphis*	Coastal/pelagic	1				
*Eubalaena australis*	Coastal/pelagic	1	1	1		
*Megaptera novaeangliae*	Coastal/pelagic	8				
*Stenella attenuata*	Coastal/pelagic	1				
*Stenella clymene*	Coastal/pelagic	5	1		1	
*Stenella coeruleoalba*	Coastal/pelagic	3				
*Stenella frontalis*	Coastal/pelagic	10	1		1	
*Steno bredanensis*	Coastal/pelagic	3				
*Tursiops truncatus*	Coastal/pelagic	3	1	1		
*Balaenoptera acutorostrata*	Pelagic	2				
*Feresa attenuata*	Pelagic	4				
*Globicephala macrorhynchus*	Pelagic	5				
*Grampus griseus*	Pelagic	2				
*Kogia breviceps*	Pelagic	9	1		1	
*Kogia sima*	Pelagic	11	1		1	
*Lagenodelphis hosei*	Pelagic	7	1		1	
*Peponocephala electra*	Pelagic	4				
*Physeter macrocephalus*	Pelagic	4				
*Ziphius cavirostris*	Pelagic	1	1		1	
Total		159	15	7	6	2

Blank cells indicate zero positive results. One individual of *Sotalia guianensis* tested positive for both *T. gondii* and *S. neurona*; therefore, the sum of parasite-specific detections is 15, although only 14 animals were identified as infected

DNA was extracted from cetacean tissues using a commercial silica-column kit, following the manufacturer’s instructions (E.Z.N.A.^®^ Tissue DNA Kit, Omega Bio-tek, Inc., Norcross, GA, USA). Molecular identification was performed by nested PCR targeting the internal transcribed spacer 1 (ITS1) region with pan-sarcocystid primers (nPCR-ITS1). A subset of samples was additionally analyzed using nested assays targeting the small subunit ribosomal RNA gene (18S rRNA) and the mitochondrial cytochrome c oxidase subunit I gene (cox1).

Amplifications of the 18S rRNA, ITS1, and cox1 loci were performed according to the protocol described by Llano et al. (2022). For ITS1 amplification, the external primers ITS1DF (5′- TACCGATTGAGTGTTCCGGTG − 3′) and CT2c (5′- CTGCAATTCACATTGCGTTTCGC − 3′), and the internal primers JS4b (5′- AGTCGTAACAAGGTTTCCGTAGG − 3′) and ITS1DiR (5′- TTCATCGTTGCGCGAGCCAAG-3′) were used; the primers ITS1DF and ITS1DiR were designed according to Rejmanek et al. (2010). The primers employed in the nPCR-cox1 included the external primers COX1 227F25 (5’ GTTTTGGTAACTACTTTGTACCGAT 3’) and COX1-885R25 (5’ GAAATATGCACGAGTATCTACCTCT 3’), and the internal primers COX1-275F22 (5’ TGTACCCACGAATTAATGCAGT 3’) and COX1-844R21 (GTGTGCCCATACTAGAGAACC), while amplification of 18S rRNA used the primers 18S9L (5’ GGATAACCGTGGTAATTCTATG 3’) and 18S1H (5’ GGCAAATGCTTTCGCAGTAG 3’).

Amplicons were visualized by agarose gel electrophoresis, purified, and sequenced using Sanger methodology. Sequences were edited and assembled with the help of CodonCode Aligner v.4.2.1 (CodonCode Corp., MA, USA). Molecular phylogenies were inferred using MEGA11: Molecular Evolutionary Genetics Analysis version 11, based on sequence alignments performed using BioEdit, available at https://bioedit.software.informer.com. Phylogenetic trees were constructed using the Maximum likelihood method. Branch support was evaluated by bootstrap analysis with 1000 replicates.

## Results

A total of 1,103 tissue samples were analyzed by nPCR-ITS1. Tissue availability varied among stranded animals; therefore, not all tissue categories were represented in every individual. In some cases, multiple samples from the same anatomical category (e.g., different brain regions or lymph nodes) were analyzed from a single animal. Overall, 55 tissue samples yielded positive amplification, corresponding to 14 positive animals. Complete screening results for all tissues from positive animals are provided in Online Resource 1.

Three reproducible amplicon sizes (~ 450 bp, ~ 900 bp, and > 1,000 bp) were consistently observed and used as provisional proxies for species identification. Sequencing of representative tissues from each positive animal confirmed that the ~ 450 bp fragment corresponded to *T. gondii*, the ~ 900 bp fragment to an undescribed *Sarcocystis* lineage, and the > 1,000 bp fragment to *S. neurona*. All but one animal exhibited a single amplicon profile; one Guiana dolphin showed co-infection with *T. gondii* and *S. neurona* in distinct tissues (Online Resource 1).

Among the 159 cetaceans examined, 14 (8.81%; 95% CI: 4.90–14.33%) were positive for Sarcocystidae by nPCR-ITS1 (Table 1). The undescribed *Sarcocystis* lineage was detected in six animals (3.77%; 95% CI: 1.40–8.03%). ITS1 amplicons of ~ 900 bp yielded partial sequences (611–882 nt), as homopolymeric regions prevented complete reads.

BLAST analyses consistently showed highest similarity with ITS1 sequences previously reported from belugas (*Delphinapterus leucas*), sperm whales (*Physeter macrocephalus*), subantarctic fur seals (*Arctocephalus tropicalis*), and minke whales (*Balaenoptera acutorostrata*) (98.38–99.88% identity). Outside this marine mammal-associated lineage, the closest ITS1 sequences retrieved in BLAST searches corresponded to *Sarcocystis canis*, although with substantially lower similarity values. Although ITS1 provides greater discriminatory power for closely related *Sarcocystis* taxa, the limited availability of ITS1 reference sequences in public databases currently restricts broader comparative analyses for this lineage.

**Fig. 1:**
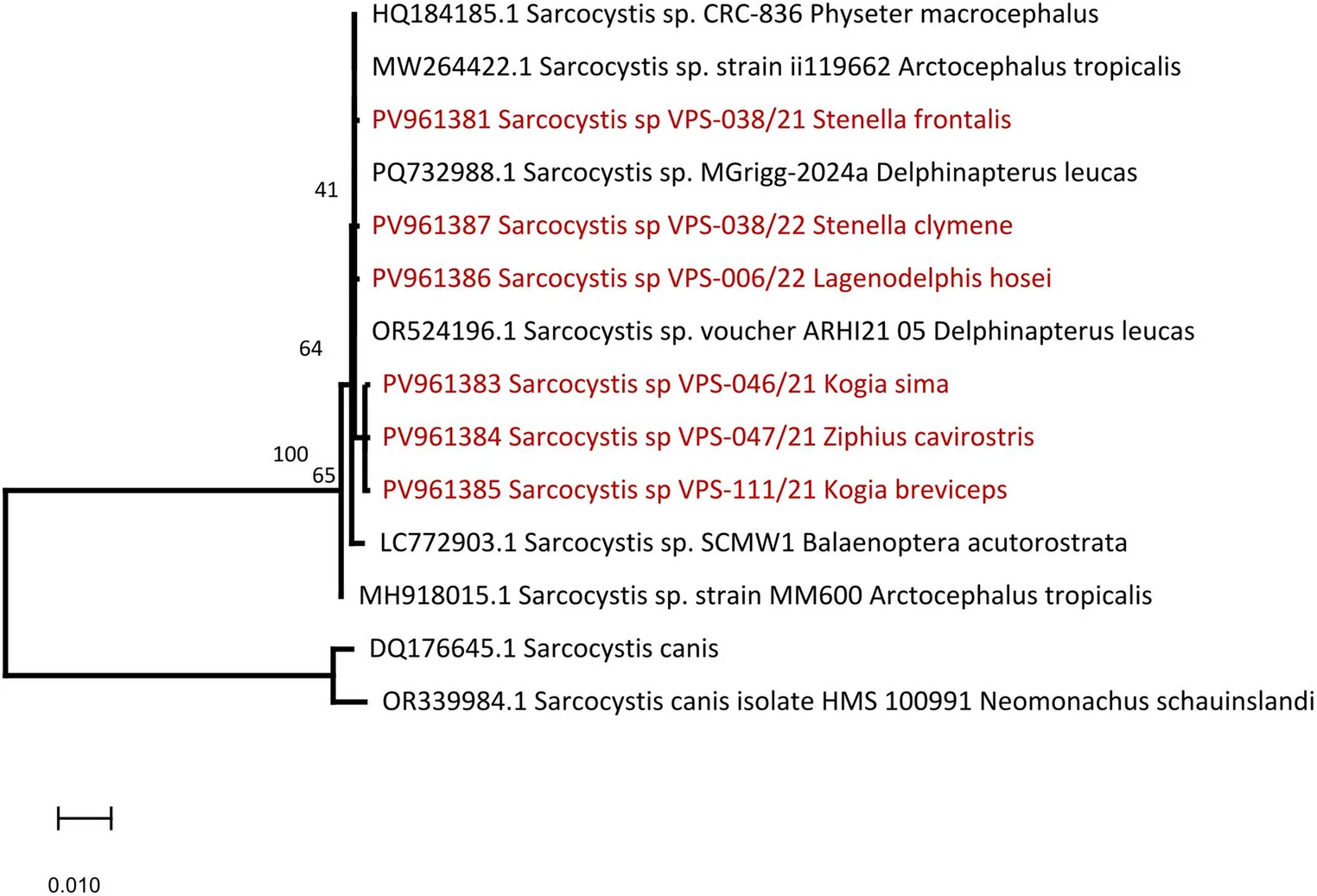
Phylogenetic relationships inferred using the Maximum Likelihood method under the Hasegawa–Kishino–Yano model. Numbers at nodes indicate bootstrap support values. The analysis included 14 nucleotide sequences and 545 aligned positions after complete deletion of gaps and missing data. Evolutionary analyses were performed in MEGA X. Sequences generated in the present study are highlighted in red. The clade corresponding to *Sarcocystis* sp. ex *Physeter macrocephalus* includes taxa previously detected in cetaceans from Japan (LC772903), Canada (PQ732988, OR524196), the United States (HQ184185), and Brazil (MW264422, MH918015)

Phylogenetic analysis based on ITS1 (Fig. 1) demonstrated that all sequences generated in the present study clustered within a strongly supported monophyletic clade together with homologous sequences previously reported from marine mammals in distinct geographic regions. Following the original designation proposed by Gibson et al. (2011), we provisionally refer to this lineage as *Sarcocystis* sp. ex *Physeter macrocephalus*, and tentatively interpret this clade as representing a single, currently undescribed species.

Additional characterization of three representative cases (46/21, 47/21, and 111/21) using 18S and cox1 markers generated identical sequences among samples (GenBank PV961388–PV961390; PV993197–PV993199). Although these loci are less informative for discriminating closely related *Sarcocystis* species, they are substantially better represented in GenBank and therefore allow broader phylogenetic comparisons. Cox1 partial sequences (547 bp) showed 99.5–100% identity with closely related species, including *Sarcocystis lutrae*, *Sarcocystis lari*, *Sarcocystis jamaicensis*, and *Sarcocystis halieti*, whereas 18S sequences (783 bp) showed 100% identity with several *S. halieti* isolates and related taxa. These findings support affinity with *Sarcocystis* species that use avian definitive hosts. Although *Sarcocystis* sp. ex *P. macrocephalus* has not yet been formally characterized using 18S or cox1 markers, one isolate from a minke whale (SCMW1; LC772902.1) exhibits an identical 18S sequence to those generated here and to *S. halieti*.

*S. neurona* DNA was identified in two Guiana dolphins (1.26%; 95% CI: 0.15–4.47%) (Online resource 1). Partial ITS1 sequences (711 bp; GenBank PV961379–PV961380) showed 100% identity with a Brazilian feline isolate (MN172273.1).

*T. gondii* DNA was detected in seven individuals (4.49%; 95% CI: 4.40–8.86%), including one co-infected with *S. neurona* (Online resource 1). ITS1 sequences (GenBank PV961373, PV961378) showed 99.8–100% identity with Brazilian bovine isolates (MH793505.1). Molecular positivity involved multiple tissues in several cases, including calves of southern right whale (*Eubalaena australis*) and Guiana dolphins.

## Discussion

The findings of this study expand the known geographic and host range of protozoan parasites detected in cetaceans stranded along the Brazilian coast. Some of these parasites are recognized pollutagens associated with land-to-sea transmission, whereas others may involve partially or exclusively marine transmission cycles.

The occurrence of *T. gondii* in Brazilian cetaceans have been documented in several species, including, Guiana dolphin, bottlenose dolphin (*Tursiops truncatus*) and Franciscana dolphin (*Pontoporia blainvillei*) (Gonzales-Viera et al. 2013; Costa-Silva et al. 2019; Sebolt et al. 2025). In this study, seven individuals were *T. gondii* positive, including five Guiana dolphins, one bottlenose dolphin, and one southern right whale. Our results therefore expand both the geographic distribution, now including northeastern Brazil, and the host range, representing the first record in southern right whale.

Importantly, the detection of infected calves may indicate early-life exposure or possible vertical transmission, reinforcing the hypothesis that these parasites can be acquired at a very young age, as previously suggested in other cetacean studies (Resendes et al. 2002). The occurrence of transplacental transmission may have important epidemiological implications, potentially contributing to parasite maintenance within cetacean populations independently of environmental exposure alone. Such a transmission route could significantly influence infection dynamics and promote distinct epidemiological patterns in marine mammal hosts.

*S. neurona* was identified in two Guiana dolphins, representing the first record in this species and in Brazilian cetaceans. The lower detection rate compared with *T. gondii* is consistent with previous studies (Gibson et al. 2011; Costa-Silva et al. 2019) and likely reflects the more restricted distribution of its definitive hosts, opossums (*Didelphis* spp.). Land-to-sea transport of sporocysts via river runoff is the most plausible transmission route, particularly in Atlantic Forest regions draining into coastal waters. Similar runoff-associated dynamics have been described for sarcocystids in marine systems, including rainfall-associated mortality events (Miller et al. 2002; Shapiro et al. 2019).

In contrast, *Sarcocystis* sp. ex *Physeter macrocephalus* displayed a distinct epidemiological pattern. Molecular analyses demonstrated high similarity to *S. halieti* and related taxa known to use raptors as definitive hosts (Prakas et al. 2025). This lineage has previously been reported in marine mammals including sperm whales, belugas, minke whales, and fur seals (Gibson et al. 2011; Reisfeld et al. 2019; Duarte-Benvenuto et al. 2022; Murata et al. 2024). Here, we expand its host range to dwarf sperm whales (*Kogia sima*), Cuvier’s beaked whales (*Ziphius cavirostris*), Fraser’s dolphins (*Lagenodelphis hosei*), Atlantic spotted dolphin (*Stenella frontalis*), and Clymene dolphins (*Stenella clymene*). Notably, infections by *Sarcocystis* sp. ex *Physeter macrocephalus* were detected exclusively in pelagic or mixed (coastal–pelagic) species, whereas *T. gondii* and *S. neurona* occurred primarily in strictly coastal dolphins, suggesting distinct transmission pathways. Its close relationship with raptor-associated *Sarcocystis* species raises the hypothesis that seabirds or coastal raptors may act as definitive hosts, shedding sporocysts into marine environments.

Infections by *S. neurona* and *T. gondii* were significantly more frequent in coastal cetaceans compared to pelagic species (9/110 vs. 0/49; Fisher’s exact test, *p* = 0.028). Their estuarine and nearshore residency (Lobo et al. 2021) likely increases exposure to terrestrial runoff, reinforcing previous evidence that coastal habitat use elevates the risk of protozoal infection. Interestingly, no sarcocystids were detected in Franciscana dolphins, despite their coastal distribution and recent reports of *T. gondii* infection in this species. Ecological or dietary differences (Di Beneditto and Ramos 2014; Campos and Santos 2021) may influence exposure risk and deserve further investigation.

Conversely, the predominance of *Sarcocystis* sp. ex *P. macrocephalus* in wide-ranging pelagic cetaceans complicates inferences regarding geographic exposure, given the extensive movements of these hosts across oceanic regions. Most pelagic species testing positive in this study, including sperm whales, Cuvier’s beaked whales, Fraser’s dolphins, and Clymene dolphins, are considered predominantly offshore and only occasional in coastal waters of the study region, reflecting broad-ranging and likely transient movement patterns. In contrast, some coastal species, such as Guiana dolphins and coastal populations of bottlenose dolphin, may exhibit greater site fidelity and prolonged use of estuarine and nearshore habitats, potentially increasing cumulative exposure to land-derived contaminants and pathogens (Santos et al. 2010). Seasonal migratory species, such as Southern right whale, may represent an intermediate ecological scenario, in which exposure could occur across multiple coastal regions during migration routes.

## Conclusions

The detection of *T. gondii* and *S. neurona* reinforces the role of terrestrially sourced pathogens in shaping marine mammal health through land-to-sea transmission processes. Both parasites are environmentally resistant and can persist in coastal ecosystems, making them useful indicators of terrestrial contamination. Meanwhile, the identification of a *Sarcocystis* lineage closely related to avian-associated species suggests more complex transmission networks potentially involving marine or coastal bird predators.

This study broadens the known host range and distribution of three sarcocystid parasites in Brazilian cetaceans. The first worldwide report of *S. neurona* in Guiana dolphins and the expansion of *Sarcocystis* sp. ex *Physeter macrocephalus* to additional pelagic hosts highlight the diversity of transmission pathways affecting marine mammals.

These findings underscore the importance of integrated One Health surveillance to clarify transmission dynamics, assess the role of vertical transmission, and evaluate the long-term impacts of protozoal infections on vulnerable cetacean populations. To this end, future studies integrating molecular detection, histopathology, immunohistochemistry, and better-preserved tissue samples will be essential to determine the pathogenic significance of these protozoa in cetacean hosts.

## Supplementary Information

Below is the link to the electronic supplementary material.

Supplementary Material 1

## Data Availability

Genetic sequences generated in this study were deposited in GenBank under accession numbers PV961373–PV961387.
