# Supplementary material for: Habitat-associated detection of Toxoplasma gondii and Sarcocystis spp. in Cetaceans from the Brazilian coast
Source: Vet Res Commun. 2026 Jun 11;50(5):383. doi: 10.1007/s11259-026-11324-y (PMC13253655; doi:10.1007/s11259-026-11324-y)
Supplement: Supplementary file 1 — Supplementary Material 1 [file 11259_2026_11324_MOESM1_ESM.docx]

Title

Habitat-Associated Detection of *Toxoplasma gondii* and *Sarcocystis* spp. in Cetaceans from the Brazilian Coast

Journal name

Veterinary Research Communications

Authors

Thalita Faita, Lara Borges Keid, Natalia Silvestre-Perez, Gláucia Pereira de Sousa, Geovana Carolaine Ramos Thomé, Beatriz de Magalhães Ceron, Samira Costa-Silva, Cristiane Kiyomi Miyaji Kolesnikovas, Pedro Volkmer de Castilho, Fernanda Loffler Niemeyer Attademo, Fábia de Oliveira Luna, Angelica María Sánchez-Sarmiento, Raquel Beneton Ferioli, Vitor Luz Carvalho, Aline Ramos Souza, Marta Jussara Cremer, Jenyffer Vierheller Vieira, Adriana Castaldo Colosio, Milton César Calzavara Marcondes, Vanessa Lanes Ribeiro, Carolina Pacheco Bertozzi, Renata Hurtado, Caroline Freitas Pessi, João Carlos Gomes Borges, Arícia Duarte-Benvenuto, Jennifer Morossino, Paula Lima Canabarro, José Luiz Catão-Dias, Rodrigo Martins Soares.

Corresponding author

Rodrigo Martins Soares. Departamento de Medicina Veterinária Preventiva e Saúde Animal, Faculdade de Medicina Veterinária e Zootecnia, Universidade de São Paulo. Av. Duque de Caxias Norte 225, 13635-900, Pirassununga, São Paulo, Brasil. Phone number +551935654296. rosoares@usp.br.

Supplementary Table. Summary of cetaceans positive for Sarcocystid apicomplexans including biological data, stranding details, sampled tissues and molecular identification

| Case | Host species and | Code | Sex | Age class | Stranding location (Beach, Municipality, State, Latitude, Longitude) | Date of stranding (condition at stranding) | nPCR-ITS1 tested tissues (positives in bold, sequenced, underlined) | Molecular identification  Genbank accession number |
| --- | --- | --- | --- | --- | --- | --- | --- | --- |
| 038/21 | Atlantic spotted dolphin (*Stenella frontalis*) | 2 | M | Adult | Ubatuba Beach, São Francisco do Sul, SC  -26.2175, -48.5214 | 20/08/2020 (dead) | ICT, HRT, LVR, MSL, **MUS, BST** | *Sarcocystis* sp*.* ex *Physeter macrocephalus*  PV961381 |
| 046/21 | Dwarf sperm whale (*Kogia sima*) | 3 | M | Adult | Guarda do Embaú Beach, Paulo Lopes, SC  -27.9400, -48.6184 | 25/05/2020 (dead) | ADR, **HRT**, DIA, LVR, **GON**, **SIT**, **LIT**, TNG, **SPC, MUS**, PAN, LNG, KDN | *Sarcocystis* sp*.* ex *Physeter macrocephalus*  PV961383 |
| 047/21 | Cuvier's beaked whale (*Ziphius cavirostris*) | 4 | M | Adult | Guarda do Embaú Beach, Paulo Lopes, SC  -27.9400, -48.6184 | 14/02/2020 (dead) | SPL, **HRT, DIA,** LVR, GON, **TNG**, LNG, KDN, CNS | *Sarcocystis* sp*.* ex *Physeter macrocephalus*  PV961384 |
| 111/21 | Pygmy sperm whale (*Kogia breviceps*) | 2 | F | Adult | Ilha Comprida Beach, Ilha Comprida, SP  -24.8038, -47.6303 | 24/11/2020 (alive) | ADR, SPL, HRT, **DIA**, LVR, **MSL**, PSL, PUL, **TNG**, **SPC**, MUS, LNG, **KDN**, **BST**, UTE | *Sarcocystis* sp*.* ex *Physeter macrocephalus*  PV961385 |
| 006/22 | Fraser dolphin (*Lagenodelphis hosei*) | 2 | F | Adult | Boracéia Beach, São Sebastião, SP  -23.7618, -45.8014 | 25/08/2021 (dead) | SPL, FTC, LVR, **PSL**, SPC, LNG, UTE | *Sarcocystis* sp*.* ex *Physeter macrocephalus*  PV961386 |
| 038/22 | Clymene dolphin (*Stenella clymene*) | 2 | M | Adult | Tabuba Beach, Caucaia, CE  -3.6595, -38.6819 | 25/04/2018 (alive) | SPL, CER, **HRT**, LVR, MSL, PUL, SPC, LNG, KDN | *Sarcocystis* sp*.* ex *Physeter macrocephalus*  PV961387 |
| 024/22 | Guiana dolphin  (*Sotalia guianensis*) | 2 | M | Adult | Cedro Beach, Ubatuba, SP  -23.4596, -45.0348 | 24/11/2021 (dead) | ADR, SPL, HRT, **FTC**, DIA, **LVR**, SIT, LIT, MSL, **PSL**, PUL, TNG, **SPC**, MUS, LNG, KDN | *Sarcocystis neurona*  PV961380 |
| 001/22* |  | 3 | M | Adult | Maranduba Beach, Ubatuba, SP  -23.5355, -45.2270 | 30/11/2021 (dead) | ADR, SPL, **CRB**, HRT, **DIA**, LVR, SIT, LIT, MSL, PSL, TNG, SML, SPC, **MUS**, PLA, **LNG**, KDN, **BST** | *Sarcocystis neurona,*  PV961379  *Toxoplasma gondii*  PV961373 |
| 007/23 |  | 3 | F | Juvenile | Baia Norte Beach, Biguaçu, SC  -27.4487, -48.6314 | 06/06/2021 (dead) | **HRT, DIA, LVR, GON, TNG, LNG, KDN, CNS, UTE** | *Toxoplasma gondii*  PV961375 |
| 011/23 |  | 3 | F | Calf | Pecém Beach, São Gonçalo do Amarante, CE  -3.5450, -38.8097 | 18/02/2021 (dead) | SPL, **HRT, FTC, LVR, MSL, LNG, KDN** | *Toxoplasma gondii*  PV961376 |
| 002/23 |  | 2 | M | Juvenile | Saco da Capela Beach, Ilhabela, SP  -23.7827, -45.3582 | 07/03/2021 (alive) | SPL, **CER, HRT, MDL, TNG, LNG, KDN** | *Toxoplasma gondii*  PV961374 |
| 124/24 |  | 2 | F | Adult | Barra dos Coqueiros, SE  -10.9081, -37.0396 | 17/06/2019 (dead) | **HRT,** KDN, LVR, LNG, LND, MUS, OVA, SPL, UTE | *Toxoplasma gondii* |
| 043/21 | Bottlenose dolphin (*Tursiops truncatus*) | 3 | M | Adult | Lagoa do Imaruí Beach, Imbituba, SC  -28.3154, -48.7388 | 08/08/2019 (dead) | ADR, SPL, CER, **HRT**, DIA, LVR, LES, MSL, **TNG**, PAN, LNG, KDN | *Toxoplasma gondii*  PV961377 |
| 128/24 | Southern right whales (*Eubalaena australis*) | 3 | M | Calf | Barra de Ibiraquera Beach, Imbituba, SC  -28.1537, -48.6507 | 21/08/2018 | **SPL,** HRT, LVR, GON, **MSL**, TNG, **KDN** | *Toxoplasma gondii*  PV961378 |

*Sample 001/22 showed co-infection with *T. gondii* and *S. neurona*. Code: carcass condition codes; 2: freshly dead; 3: moderately decomposed but tissues largely intact; 4: advanced decomposition. In sample 001/22, MUS, BST and LNG were positive for *T. gondii*, whereas CRB and DIA were positive for *S. neurona.* ADR: adrenal gland. BST: brain stem. CER: cerebrum. CNS: central nervous system. CRB: cerebellum. CTX: cortex. DIA: diaphragm. FTC: frontal cortex. GON: gonad. HRT: heart. ICT: intestinal content. KDN: kidney. LIT: large intestine. LNG: lung. LND: lymph node (unspecified). LVR: liver. MDL: mediastinal lymph node. MSL: mesenteric lymph node. MUS: muscle. OVA: ovary. PAN: pancreas. PEN: penis. PLA: placenta. PSL: prescapular lymph node. PUL: pulmonary lymph node. RCL: rectal lymph node. SIT: small intestine. SKN: skin. SML: submandibular lymph node. SPC: spinal cord. SPL: spleen. STM: stomach. TNG: tongue. UTE: uterus.
